# Vaccinations and Healthy Ageing: How to Rise to the Challenge Following a Life-Course Vaccination Approach

**DOI:** 10.3390/vaccines10030375

**Published:** 2022-02-28

**Authors:** Francesca Scognamiglio, Maria Pia Fantini, Chiara Reno, Marco Montalti, Zeno Di Valerio, Giorgia Soldà, Aurelia Salussolia, Giusy La Fauci, Angelo Capodici, Davide Gori

**Affiliations:** 1 School of Hygiene and Preventive Medicine, Department of Biomedical and Neuromotor Sciences, Public Health and Medical Statistics, University of Bologna, Via San Giacomo 12, 40126 Bologna, Italy; chiara.reno@studio.unibo.it (C.R.); marco.montalti7@studio.unibo.it (M.M.); zeno.divalerio@studio.unibo.it (Z.D.V.); giorgia.solda@studio.unibo.it (G.S.); aurelia.salussolia@studio.unibo.it (A.S.); giusy.lafauci@studio.unibo.it (G.L.F.); angelo.capodici@studio.unibo.it (A.C.); 2 Unit of Hygiene, Department of Biomedical and Neuromotor Sciences, Public Health and Medical Statistics, University of Bologna, Via San Giacomo 12, 40126 Bologna, Italy; mariapia.fantini@unibo.it (M.P.F.); davide.gori4@unibo.it (D.G.)

**Keywords:** vaccination, healthy ageing, immune fitness, life-course vaccination

## Abstract

In the context of an ageing population, one of the major Public Health goals is to promote healthy ageing. To rise to this challenge, rethinking conventional prevention paradigms and implementing them with vaccination at all stages of life is necessary. Indeed, vaccinations are able to both prevent pathogen specific diseases and all their downstream effects and to increase overall immune system plasticity and resilience. Our aim is to discuss the obstacles and opportunities in pursuing a “life-course vaccination approach” and to highlight the role of vaccines in healthy ageing. In doing so, we address the close connections between immunology and vaccinology advances and introduce the novel concept of immune fitness. Finally, we conclude that even though vaccinology is making giant steps towards tailored vaccination strategies, more studies are needed to investigate this topic.

## 1. Introduction

Transmittable diseases have always represented a major threat, and have determined high mortality and morbidity throughout history. Over the last two centuries important public health measures such as water purification, sanitation/hygiene, and vaccinations, as well as better nutrition conditions and the use of antibiotics, have helped tackle infectious diseases, thus reducing infant mortality and morbidity and increasing general life expectancy [1].

Notably, among all the elements mentioned above vaccines are second only to water purification in terms of mortality reduction, making immunization a global public health success that currently prevents 4–5 million deaths each year [2]. Indeed, the introduction of mass immunization campaigns has determined a sharp decrease in vaccine-preventable disease (VPD) incidence in high-, low-, and middle-income countries, thus reducing the burden of infectious diseases. Thanks to mass vaccination campaigns, smallpox was eradicated in 1979, five of the six World Health Organization (WHO) regions are currently free of wild poliovirus [3], and under-five infant mortality has decreased by 60%, from 93 deaths per 1000 live births in 1990 to 38 in 2019 [4]. Moreover, vaccinations are able to prevent the downstream effects of infectious diseases such as the increase in cardiovascular and cerebrovascular events following influenza infection [5], reactivation of the Varicella Zoster Virus (VZV) [6,7,8], and the increase in all-cause mortality rates due to the immunosuppression that follows measles infection [9]; they therefore constitute an indispensable preventative tool for the health and well-being of the population.

Despite the success of vaccination, the global burden of communicable diseases remains immense, and affects mostly the youngest and the oldest, especially in middle- and low- income countries. In 2019 alone, 7.4 million children, adolescents and youths (0–14 years) died, mostly of preventable or treatable causes [4]. Indeed, lower respiratory tract infections, diarrhoeal diseases, meningitis, whooping cough, tuberculosis, malaria, and HIV/AIDS represent several of the most significant causes of disability-adjusted life-years (DALYs) worldwide in people under 24 years of age [10], whereas lower respiratory tract infections, diarrhoeal diseases, and tuberculosis determine a high burden of disease in people older than 50 years of age, along with chronic degenerative diseases [10].

This incredibly complex scenario has been further complicated by the current COVID-19 pandemic, which has once again highlighted the tremendous impact of infectious diseases in terms of public health as well as the importance of vaccinations to tackle them and protect the most fragile groups, such as the elderly and people with comorbidities [11,12].

Following these considerations, today one of the major challenges of public health systems is to ensure adequate vaccination coverage and promote vaccination at all stages of life (referred to here as life-course vaccination) [6,13,14,15,16].

This paper aims to discuss obstacles and opportunities in pursuing a “life-course vaccination approach” by highlighting the role of vaccines in healthy ageing. An important premise is to describe new technologies in vaccinology and the connection between vaccines and immunological responses, introducing the novel concept of immune fitness [17].

In this review, we discuss how new technologies in the field of vaccinology have enabled the development of vaccines capable of eliciting an adequate immune response even in the elderly and in individuals with a compromised immune system, thus making it possible to vaccinate at all stages of life (a life-course vaccination approach).

In addition, we explore how new discoveries in the field of immunology have made it possible to both identify new targets for vaccines and to define the impact that vaccinations have on the immune system of individuals, increasing its plasticity and resilience recently defined as “immune fitness” in a paper by Laupèze et al. [17].

## 2. How to Rise to the Challenge of a “Life-Course Vaccination Approach”: Vaccinology and Its Novel Advances

Vaccination is now in its third century of practice. Beginning in 1796, the year in which vaccinology was born thanks to Edward Jenner, different approaches have implemented vaccine features in terms of effectiveness and safety [18].

Until 1980, an empirical approach was used and microorganisms or their toxins had to be attenuated or killed and injected into the vaccine in order to elicit an immune response in the recipient. This method enabled the development of vaccines that, while generally highly immunogenic, had several side effects due to their high reactogenicity. Rabies vaccine, Bacillus Calmette–Guérin (BCG), the Diphtheria and Tetanus vaccines, the Mumps, Rubella, and Measles vaccines, live attenuated and killed Polio vaccines, and polysaccharide vaccines against Meningococcus A, C, W, Y, and Haemophilus influenzae type B were all obtained by following an empirical approach [18].

In the 1980s, important technological advances led to the production of conjugated vaccines and vaccines obtained using recombinant DNA technology.

Glycoconjugate vaccines addressed the problem of low immunogenicity in highly purified vaccines by conjugating polysaccharides to proteins, leading to the production of novel conjugated vaccines against Pneumococcus, Haemophilus Influenzae type B, and Meningococcus A, C, W, and Y which were able to elicit a better immunological response in both children and in the elderly [18].

Recombinant DNA technology provided the ability to produce pathogen components in non-pathogenic vectors without the need for the pathogen itself, improving the safety and purity of vaccines and enabling the development of vaccines against Hepatitis B and Human Papilloma Virus, among others [18].

In recent years, the availability of wide genomic screening has given rise to the possibility of analysing entire bacterial and viral genomes to discover novel vaccine targets. This new approach, known as “reverse vaccinology”, has led to the development of vaccines otherwise impossible to produce, such as the Meningococcus B vaccine.

Moreover, other innovative and interesting approaches include synthetic biology and structural biology. While synthetic biology allows the design of artificial molecules such as DNA and RNA, structural biology studies the structure and function of proteins [18]. These technologies have recently gained prominence due also to their application in the development of several COVID-19 vaccines [19]. Both approaches aim to design specific antigenic targets able to elicit a desired immunological response, opening the way to a new era of “tailored vaccinology”.

From this point of view, another fundamental step is adjuvant research. Adjuvants are molecules that are able to improve the immunogenicity of vaccines and modulate the immune response in the desired way [18]. They have led to the development of vaccines able to elicit an adequate immune response with highly purified antigens and made possible their use in individuals with a poorly-functioning immune system, such as the elderly (whose immune system undergoes a functional decline defined as “immunosenescence”) and people with comorbidities. In the context of an ageing population and in order to follow a “life-course vaccination approach”, adjuvant research and tailored vaccinology are fundamental tools to guarantee adequate immunization rates and promote the health of the population.

## 3. Immunosenescence and Inflammaging: Description of an Ageing Immune System

As mentioned above, life expectancy has been increasing over the last several decades and the global population is ageing; an estimated one person out of every six will be aged 60 years or older by 2050 [20]. In addition to the increasing burden of non-communicable diseases, infectious diseases remain responsible for morbidity and mortality among older adults due to the functional decline of both their innate and adaptive immune systems, defined as “immunosenescence” [21,22,23]. In older people, the primary lymphoid organs (thymus and bone marrow) involute, causing a decrease in the number of naive B-cells and T-cells. Moreover, functional alterations and specific epigenetic patterns with detrimental effects on immune response become increasingly present.

Another major feature of the senescent immune system is the presence of a low-grade proinflammatory status called “inflammaging” provoked by continuous antigenic load and stress [24]. The persistence of this pro-inflammatory status represents the first step in developing age-related diseases, and accelerates the ageing process itself.

Overall, these changes determine the impaired capacity of response to antigenic stimuli as well as the ability to tackle infections, and develop adequate immune responses to vaccines. Currently, new research lines are opening innovative routes to find a solution to this complex phenomenon. One area of potential interest is the use of monoclonal antibodies to target inhibitory receptors such as CTLA-4 and PD-1 in order to enhance T-cell response and the overall immunogenicity of vaccines [22]. Anti-inflammatory drugs and immunomodulators able to block baseline inflammation status (m-TOR inhibitors, metformin, imiquimod, and COX2-inhibitors) may provide a valuable weapon against inflammaging, and could help tackle the impaired immunogenicity that accompanies the ageing process [22,25,26]. One of the most promising approaches lies in the stimulation by vaccination of cross-reactive T-cells, which can recognize highly-conserved peptides and thus induce heterosubtypic immunity, which provides broader protection against pathogenic threats [21,22]. Other interesting and innovative lines of research aim to develop vaccines capable of triggering both the adaptive immune memory and a mechanism of medium to long term innate immunological memory recently defined as ‘trained immunity’, which we discuss in more detail in the following section [27,28,29].

## 4. Trained Immunity and the Role of Adjuvants

When considering new strategies to enhance immunogenicity, the importance of adjuvant substances must be highlighted. Vaccine adjuvants are molecules or compounds that have intrinsic immunomodulatory properties and which, when administered in conjunction with an antigen, potentiate the host’s antigen-specific immune responses more effectively compared to antigen response alone [30]. Moreover, they are able to enhance vaccine immunogenicity, acting at different levels due to their capacity to activate innate immune responses and polarize the adaptive immune response [22].

In the context of the current vaccinology paradigm (towards the use of extremely purified, less immunogenic antigens), adjuvants are essential to achieve adequate immunogenicity and vaccine effectiveness in every age, and particularly in older people affected by immunosenescence. Whereas early adjuvants such as aluminium salts were used empirically, better understanding of the mechanisms underlying immune system function are the starting point for modern research lines that can be better tailored in order to achieve adequate immunogenicity and limit side effects [31].

Currently only two adjuvants (MF59 and AS01B) are licensed for individuals older than 65 years of age [22]. Promising research pathways involve the use of adjuvants that contain Pathogen-Associated Molecular Patterns (PAMPs) and are therefore able to determine broader protection by stimulating cross reactive T-cells, as well as adjuvants that can stimulate Toll-Like Receptors (TLR), thus promoting cytokine production and stimulating innate and adaptive responses at the same time. In this way, novel adjuvants should be able to trigger innate trained immunity, a form of innate immune memory with a key role in achieving pathogen protection [22]. Indeed, recent evidence has demonstrated that antigen stimulation can lead to metabolic changes in the cells responsible for innate immunity, resulting in different regulation at the epigenetic level, which in turn leads to changes in the expression of innate immunity receptors that can last for a long period of time [17,22]. In addition to the adaptive immune response being better stimulated by adjuvants, trained immunity allows the innate immune system the ability to elicit a better immunological response against unrelated antigenic threats, and could explain the “paraimmunity” phenomenon associated with vaccination. For instance, a meta-analysis showed that all-cause mortality in children was reduced by 49% after administration of measles vaccination [17,32], and studies have found a correlation between BCG vaccination and reduced COVID-19 mortality [17,33]. In addition, the EPI-ZOSTER-089 study assessed whether recombinant herpes zoster vaccination (RZV) resulted in a lower risk of COVID-19 [34]. The study involved two approaches, namely, a matched cohort and a test negative approach, in order to reduce the risk of bias; the two approaches provided consistent results. In particular, vaccination with at least one dose of RZV was associated with a 16% lower rate of diagnosis of COVID-19 and a 32% lower rate of hospitalisation. Vaccination with two doses of RZV was associated with a 19% lower rate of COVID-19 diagnosis and a 36% lower rate of hospitalisation (after adjusting for BMI, smoking habit, number of outpatient visits, hypertension, and other vaccination-related covariates) [34].

In summation, by triggering trained immunity, novel vaccine adjuvants seem to be able to overcome immunosenescence and improve the performance of the immune system in immunised individuals.

## 5. Tailored Vaccinology as a Way to Overcome Immunosenescence

In the previous sections, we discussed immunosenescence and the most promising lines of research in vaccinology. Here, we will review the current vaccination strategies that public health authorities are adopting to overcome the problem of immunosenescence and obtain adequate immunisation rates in the older population following the paradigm of a life course vaccination approach. The recent progress that has been made in implementing vaccination strategies specific to this target population will be reviewed as well.

Presently, several vaccines are recommended for older adults, including the influenza, herpes zoster (HZV), and pneumococcal vaccines. In detail, adjuvanted influenza vaccines and high antigen dose influenza vaccines are targeted towards people older than 65 years of age because of their higher immunogenicity compared with other influenza vaccines [22,35].

For HZV, the recent introduction of adjuvanted recombinant herpes zoster vaccine has dramatically improved vaccine efficacy compared to live attenuated zoster vaccines. Indeed, whereas live attenuated varicella zoster vaccine presented age-dependent vaccine efficacy, dropping from 64% in the 60–69 age group to 41% in the 70–79 age group, herpes zoster adjuvanted recombinant vaccine has demonstrated elevated and age-independent vaccine efficacy [22,36,37], corroborated by the previous reported evidence of the possible protection against severe COVID-19 symptoms [34].

Until novel and more effective vaccination strategies are developed, it is crucial to promote and achieve high vaccination coverage in the adult population. Indeed, we cannot forget that adult vaccine coverage is almost always lower than paediatric vaccine coverage in the same country, even where vaccinations are fully reimbursed and free at point of care, because of a cultural bias that makes people perceive vaccination as a “paediatric issue”.

Therefore, a cultural paradigm shift in vaccination perception is necessary, in addition to all the other necessary changes in terms of accessibility, affordability, infrastructure, and reimbursement [6].

## 6. From the Definition of Immune Biography to the Definition of Immune Fitness

We have discussed the burden of infectious diseases and the importance of offering vaccinations at all ages and stages of life, promoting a life-course approach to vaccination. Following this approach, it is possible to reduce morbidity and mortality caused by communicable diseases and prevent all of their down-stream effects (such as enhanced cardiovascular and cerebrovascular events) and thereby promote healthy ageing and good health conditions [38].

Diet, exercise, and smoking are well-known environmental factors capable of having an impact on the immune system. As with every other stimulus, vaccination shapes the immune system, enhancing pathogen specific protection together with immune system plasticity and resilience, which is defined as “immune fitness” in one study by Laupèze et al. [17]. This novel concept of immune fitness indicates the ability of an individual’s immune system to elicit an effective immune response to external stimuli (defined as resilience) and to return to baseline conditions after extinction of the stimulus, thereby maintaining proper homeostasis [17]. From this perspective, the life course vaccination approach in combination with known healthy life-styles could be a fundamental preventative tool.

## 7. Conclusions

Today, public health systems face the enormous challenge of promoting healthy ageing of the population. In order to rise to this challenge, prevention is mandatory; however, a paradigm shift is needed as well. In addition to diet, physical exercise, and smoking cessation, vaccines must become part of the tools available for health promotion by following a life-course vaccination approach. Indeed, in discussing the obstacles and opportunities of this novel approach to vaccination we have seen how vaccinations are crucial both to preventing infectious diseases and to training the plasticity and resilience of the immune system. Moreover, we have looked at how advances in immunological knowledge and vaccinology are enabling the emergence of new vaccination strategies tailored to specific target populations, such as the elderly and individuals with comorbidities. Although much progress has been made, further studies are needed in this field to move towards increasingly refined and tailored strategies.

## Data Availability

Not applicable.
